# IGF-1 Promotes Brn-4 Expression and Neuronal Differentiation of Neural Stem Cells via the PI3K/Akt Pathway

**DOI:** 10.1371/journal.pone.0113801

**Published:** 2014-12-04

**Authors:** Xinhua Zhang, Lei Zhang, Xiang Cheng, Yuxiu Guo, Xiaohui Sun, Geng Chen, Haoming Li, Pengcheng Li, Xiaohui Lu, Meiling Tian, Jianbing Qin, Hui Zhou, Guohua Jin

**Affiliations:** 1 Department of Anatomy, Nantong University, Nantong, Jiangsu, China; 2 Department of Pediatrics, Affiliated Hospital of Nantong University, Nantong, Jiangsu, China; 3 Vasculocardiology Department, Nantong Rehibilitation Hosptital Agings, Nantong, Jiangsu, China; 4 Department of Stomatology, Affiliated Hospital of Nantong University, Nantong, Jiangsu, China; Temple University School of Medicine, United States of America

## Abstract

Our previous studies indicated that transcription factor Brn-4 is upregulated in the surgically denervated hippocampus *in vivo*, promoting neuronal differentiation of hippocampal neural stem cells (NSCs) *in vitro*. The molecules mediating Brn-4 upregulation in the denervated hippocampus remain unknown. In this study we examined the levels of insulin-like growth factor-1 (IGF-1) in hippocampus following denervation. Surgical denervation led to a significant increase in IGF-1 expression *in vivo*. We also report that IGF-1 treatment on NSCs *in vitro* led to a marked acceleration of Brn-4 expression and cell differentiation down neuronal pathways. The promotion effects were blocked by PI3K-specific inhibitor (LY294002), but not MAPK inhibitor (PD98059); levels of phospho-Akt were increased by IGF-1 treatment. In addition, inhibition of IGF-1 receptor (AG1024) and mTOR (rapamycin) both attenuated the increased expression of Brn-4 induced by IGF-1. Together, the results demonstrated that upregulation of IGF-1 induced by hippocampal denervation injury leads to activation of the PI3K/Akt signaling pathway, which in turn gives rise to upregulation of the Brn-4 and subsequent stem cell differentiation down neuronal pathways.

## Introduction

Degeneration, necrosis, or loss of neurons is pathological characteristics of many nervous system diseases. Replacement of the lost neurons by transplantation of exogenous neurons, or by activation of endogenous neurons or their precursors, may provide a treatment for nervous system diseases. Previous studies have shown that neural stem cells (NSCs) are present not only in embryonic brain tissue but also in the adult dentate gyrus of the hippocampus and subventricular zone [1], [2]. These cells possess stem cell properties including self-renewal, proliferation, and multipotent differentiation. NSCs are therefore generally considered to be a potential source of cells for cell replacement therapy. However, NSCs only produce a small number of neurons under normal conditions. Some external factors such as NG2 and Mash1 [3], [4], [5] can promote NSC differentiation into neurons, but the numbers of differentiated neurons remain too low to meet treatment demands. It is therefore important to identify the factors and mechanisms involved in neuronal differentiation of NSCs to guide the production of NSCs for clinical needs.

We previously reported that the environment of the denervated hippocampus following fimbria fornix (FiFx) transection significantly improved the survival, migration, and neuronal differentiation of both grafted and endogenous newborn NSCs compared with normal hippocampus [6], [7]. These results indicated that the denervated hippocampus provides a microenvironment suitable for the survival and differentiation of NSCs. It is therefore important to determine the cues in the denervated hippocampus that are responsible for this phenomenon. We previously reported that Brn-4, a member of the POU-III class of transcription factors [8], is upregulated in the hippocampus after denervation surgery [9]. Previous studies showed that POU genes display cell type-specific gene expression in mammals [8], [10], [11], [12], [13]. Transcription in NSCs is regulated by a combination of POU-domain factors [14] and we previously presented evidence that upregulation of Brn-4 is involved in the differentiation of NSCs into neurons [9], [15]. Shimazaki *et al.* [16] confirmed that exposure of NSCs derived from embryonic (E) day E14 mouse striatum to either insulin-like growth factor-1 (IGF-1) or brain-derived neurotrophic factor (BDNF) resulted in rapid upregulation of Brn-4 mRNA and protein levels; upregulation was accompanied by increased neuronal differentiation which could be attenuated by Brn-4 antisense oligonucleotides.

These results suggest that Brn-4 plays an important role in driving neuronal differentiation of NSCs, and that its expression is subject to growth factor control. However, the molecular mechanisms underlying Brn-4 upregulation and/or Brn-4-mediated neuronal differentiation of NSCs remain unknown. In the present study we wished to determine whether IGF-1 is involved in the upregulation of Brn-4 expression and neuronal differentiation taking place in the hippocampus following denervation. We therefore investigated potential changes in IGF-1 levels in the denervated hippocampus *in vivo*, and also whether IGF-1 influences Brn-4 expression and neuronal differentiation of hippocampus-derived NSCs *in vitro*. This work led to the identification of a molecular pathway controlling Brn-4 expression. The results of this study provide a theoretical basis for inducing neuronal differentiation of hippocampal NSCs and facilitating the development of NSCs for clinic use.

## Materials and Methods

### 1. Reagents

Dulbecco’s modified Eagle’s medium/F12 (1∶1, DMEM/F12,) and B27 were from Gibco (Grand Island, NY, USA). Epidermal growth factor (EGF), basic fibroblast growth factor (bFGF), trypsin, and dimethyl sulfoxide (DMSO) were purchased from Sigma (St Louis, MO, USA). IGF-1, PD98059, and LY294002 were from Invitrogen (Carlsbad, CA, USA). Both PD98059 and LY294002 were dissolved in DMSO and stored at 50 mM. Rapamycin was from Beyotime (Nantong, CHN). AG1024 was form Selleck (Housten, TX, US). Other reagents are described below.

### 2. Animals and surgery

All animal experiments were carried out in accordance with the United States National Institutes of Health Guide for the Care and Use of Laboratory Animals. The study protocol was approved by the Care and Use committee of Laboratory Animal Research Center of Nantong University. All efforts were made to minimize the number and suffering of animals used in this study. Adult female Sprague-Dawley rats weighing 200–250 g and pregnant Sprague-Dawley rats were purchased from the experimental animal center of Nantong University. Transection of the right FiFx was performed as described previously [7]. After transection, rats were caged in an approved facility with *ad libitum* access to food and water. Seven days later, the rats were anesthetized with chloral hydrate (2 ml/kg body weight, delivered intraperitoneally), and perfused with 0.9% (w/v) NaCl and 4% (w/v) paraformaldehyde in 0.1 M phosphate-buffered saline (PBS). Coronal sections (20 µm) of the lesioned site or hippocampus were prepared using a Leica cryostat (Leica CM1900, Solms, Germany). Nissl staining (0.1% cresyl violet) [54] and immunofluorescence assays were used to examine the success of the FiFx lesion model. For IGF-1 administration, IGF-1 (0.5 µg/100 g body weitht) was injected to the right side hippocampus of the rat (coordinates: 3.0 mm caudal and 2.0 mm right from bregma; 3.0 mm deep). The injection was completed in 5 min. Then the needle was kept in the position for additional 3 min and retrieved slowly out of the brain. Three days later, coronal sections (20 µm) of the hippocampus were prepared as described previously.

### 3. NSC culture

NSCs were derived from the hippocampus of E14 rats as described previously [6], [9], [55]. In brief, after anesthesia with chloral hydrate (2 ml/kg body weight), the hippocampus was quickly dissected, digested with 0.125% trypsin, and then dissociated mechanically into single-cell suspensions. The cell suspensions were plated into flasks at a density of 1×10^4^ cells/ml with NSC culture medium (1∶1, DMEM/F12) containing 2% B27, 10 ng/ml EGF, 10 ng/ml bFGF, 100 U/ml penicillin/streptomycin, and maintained in a humidified 95% air 5% (v/v) CO_2_ incubator at 37°C. After 5 days, neurospheres were dissociated into single-cell suspensions and seeded in 96-well plates at 1–2 cells per well. The subclonal neurospheres were then digested and passaged again as before. Cells were passaged three times to obtain neurospheres originating from a single primary cell. After digestion of the neurospheres, the NSCs were seeded into multi-well plates at a density of 5×10^5^ cells/ml for subsequent experimentation. For neuronal differentiation, NSC culture medium was replaced by differentiation medium (DMEM +2% fetal bovine serum, FBS) and incubation continued as before.

### 4. Immunofluorescence assay

Cells seeded in 24-well plates were washed twice with ice-cold PBS, fixed with 100% methanol for 7 min at –20°C, and permeated with fresh 4% paraformaldehyde for 20 min at room temperature. These cells and the coronal cryostat sections through the hippocampi were blocked with blocking buffer (10% goat serum in PBS containing 0.3% Triton X-100 and 0.03% NaN_3_) overnight at 4°C. The cells and sections were then incubated with the primary antibody diluted in blocking buffer at 4°C for 24 h, followed by incubation with the secondary antibody overnight at 4°C. After further washing with PBS, the cells and sections were stained with Hoechst (1∶1,000; Pierce, Rockford, IL, USA) for 0.5 h at room temperature, and then viewed under a fluorescence microscope (Leica DMIRB, Germany). Primary antibodies were as follows: mouse monoclonal anti-choline acetyltransferase (ChAT) (1∶500, Abcam, Cambridge, MA, USA), rabbit polyclonal anti-Brn-4 (1∶200, Santa Cruz, CA, USA), mouse monoclonal anti-microtubule-associated protein (MAP)-2 (1∶1,000; Millipore, Billerica, MA, USA) and rabbit polyclonal anti-Brn4 (1∶500; Santa Cruz, CA, USA). Secondary antibodies were: Alexa Fluor 568-conjugated (red) goat anti-mouse IgG (1∶500; Invitrogen, Carlsbad, CA, USA), and Alexa Fluor 488-conjugated (green) goat anti-rabbit IgG (1∶200; Invitrogen, Carlsbad, CA, USA).

### 5. Western blotting analysis

Seven days after right FiFx transection, rats were anesthetized, killed by cervical dislocation, and hippocampi removed. Proteins from hippocampal tissue proteins and from NSCs seeded in the 6-well plates were extracted using a mammalian Protein Extraction Reagent (Pierce, Rockford, IL, USA) according to the manufacturer’s instructions. The protein contents were determined using an Enhanced BCA (bicinchoninic acid) Protein Assay kit (Beyotime, Jiangsu, CHN). Equivalent amounts of protein (30 µg) were loaded into each well and separated on 20% polyacrylamide gels in the presence of sodium dodecyl sulfate (SDS) to detect IGF-1, or on 10% gels to detect other proteins, then transferred to polyvinylidene difluoride membranes (Bio-Rad, Hercules, CA, USA). Membranes were subsequently blocked with 5% non-fat milk in TBS and incubated overnight at 4°C with mouse monoclonal anti-IGF-1 (1∶1,000, Sigma, St Louis, MO, USA), rabbit polyclonal anti-Brn-4 (1∶1,000, Santa Cruz, CA, USA), mouse monoclonal anti-β-actin (1∶10,000, Sigma, St Louis, MO, USA), rabbit polyclonal anti-Akt or anti-phospho-Akt (1∶1,000, Beyotime, Jiangsu, CHN), rabbit polyclonal anti-mitogen-activated protein kinase (MAPK) and phospho-MAPK (1∶1,000, Beyotime, Jiangsu, CHN). Membranes were developed by incubation with horseradish peroxidase-conjugated goat anti-mouse or rabbit IgG (1∶3,000, Pierce, Rockford, IL, USA) for 2 h at room temperature. After washing, the complexes were visualized by enhanced chemiluminescence (Santa Cruz, CA, USA) and exposed to X-ray film (Kodak, Rochester, NY, USA). The intensity of each band was quantified using the Shine-tech Image System (Shanghai, CHN).

### 6. Reverse transcription-polymerase chain reaction (RT-PCR) analysis

Total RNA was extracted from the treated cells and the hippocampi of FiFx transected rats using a Trizol reagent kit (BBI, CAN). In each case 2 µg of total RNA were reverse transcribed into cDNA using oligo(dT) primers and Omniscript reverse transcriptase (Qiagen, Hilden, GER) according to the manufacturer’s instructions. PCR was performed using the following primers (synthesized by Sangon, Shanghai, CHN): IGF-1: forward, 5′-CAG TTC GTG TGT GGA CCA AG-3′, reverse, 5′-GTC TTG GGC ATG TCA GTG TG-3′; Brn-4: forward, 5′-GGG TGA CCA GTC TTA GCG AC-3′, reverse, 5′-GCG AGT ACA CAT TGA GGG GT-3′; glyceraldehyde 3-phosphate dehydrogenase (GAPDH): forward, 5′-ACCACAGTCCATGCCATCAC-3′, reverse, 5′-TCCACCACCCTGTTGCTGTA-3′; β-actin: forward, 5′-CCCTAAGGCCAACCGTGAAAAGATG-3′, reverse, 5′-GAACCGCTCATTGCCGATAGTGATG-3′.

PCR products were separated by agarose gel electrophoresis and band densities of IGF-1 and Brn-4, relative to β-actin or GAPDH, was quantified using an image analysis system (Leica Q550I W, Solms, GER).

### 7. IGF-1 enzyme-linked immunosorbent assay (ELISA)

Seven days after FiFx transection, normal and denervated hippocampi were dissociated using an aseptic glass homogenizer in cold DMEM (1 ml/100 mg hippocampus) and homogenized for 10 min. The homogenate was then centrifuged at 4°C at 250 *g* for 5 min and the supernatant was harvested and tested using a rat IGF-1 ELISA kit (Jianglai, Shanghai, CHN) according to the manufacturer’s recommendations.

### 8. Statistical analysis

Statistical analysis was carried out using GraphPad software (GraphPad Prism v4.0, GraphPad Software, San Diego, CA, USA). Data are expressed as means ± SEM and submitted to one/two-way ANOVA followed by either, Newman-Keuls or Bonferroni’s multiple comparison test (as a *post hoc* test). *P*<0.05 was considered to indicate statistical significance.

## Results

### 1. FiFx transection induced loss of cholinergic fibers in the hippocampus

**Figure 1:**
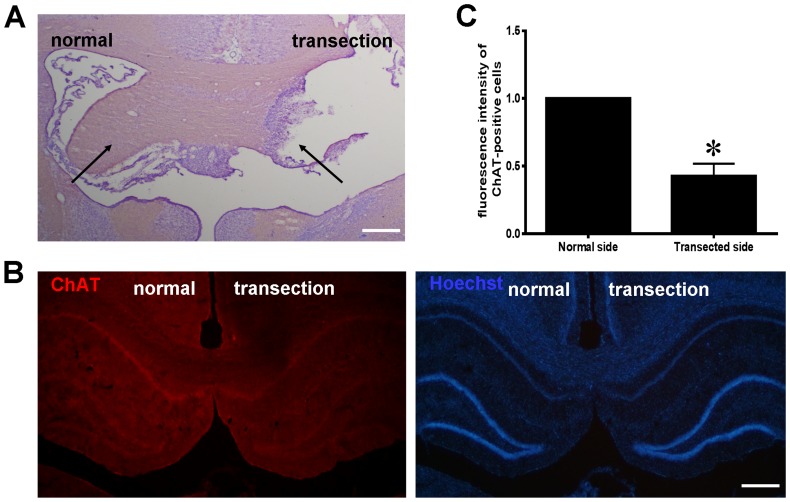
Confirmation of the FiFx transection model. (**A**) Nissl staining of a coronal section through the FiFx at day 7 after right FiFx transection. Arrows show loss of the right FiFx whereas the left FiFx remains intact (*n* = 10; a representative panel is shown). (**B**) ChAT immunofluorescence staining of coronal section through hippocampus at day 7 after right FiFx transection. (**C**) Quantification of fluorescence intensity of ChAT-positive cells in the transected or normal side. The normal side set to 1. *, *P*<0.05. Scale bar, 300 µm.

Hippocampal denervation was performed through unilateral FiFx transection, severing afferent cholinergic fibers from the septal area to the hippocampus. To assess the efficacy of denervation, sections through the FiFx and hippocampus were stained using either Nissl or ChAT immunofluorescence. Nissl staining showed that the right FiFx was completely transected whereas the left remained intact (Fig. 1A). ChAT-positive fluorescence intensity in the denervated hippocampus was significantly weaker than in the control contralateral hippocampus (Fig. 1B, C), confirming successful FiFx transection.

### 2. IGF-1 expression is increased in FiFx transected hippocampus

**Figure 2:**
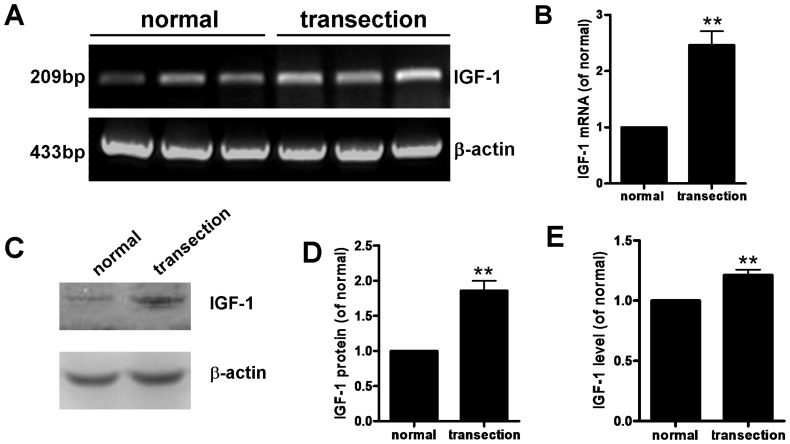
IGF-1 mRNA and protein in normal and denervated hippocampi at day 7 after right FiFx transection. (**A**) RT-PCR determination of IGF-1 and β-actin (reference) mRNA levels. (**B**) Quantification of IGF-1 mRNA levels in (A). (**C**) Levels of IGF-1 protein (7 kDa) detected by western blotting (β-actin reference, 42 kDa). (**D**) Quantification of IGF-1 protein levels in (C). (**E**) ELISA showing IGF-1 protein levels in normal and denervated hippocampi. Data are means ± s.e.m.; **, *P*<0.01 versus normal hippocampus.

On day 7 after FiFx transection, total RNA and protein were extracted from normal and denervated hippocampi; levels of IGF-1 mRNA and protein in the hippocampus after FiFx transection were determined by RT-PCR (Fig. 2A, B), western blotting analysis (Fig. 2C, D), and ELISA (Fig. 2E). RT-PCR revealed that IGF-1 mRNA levels in the denervated hippocampus were significantly greater than in the contralateral (normal) tissue (*P*<0.01, Fig. 2A, B). Western blotting and ELISA (Fig. 2C–E) both confirmed that denervation significantly increased IGF-1 in the hippocampus, and IGF-1 protein levels in denervated hippocampus determined by western blotting were more than twofold increased versus control hippocampus (*P*<0.01). Although the extent of the increase was less marked when determined by ELISA, the increase remained statistically significant (*P*<0.05). These results indicate that hippocampal denervation is accompanied by increased IGF-1 expression at both the mRNA and protein levels.

### 3. IGF-1 increases Brn-4 expression *in vivo and in vitro*

**Figure 3:**
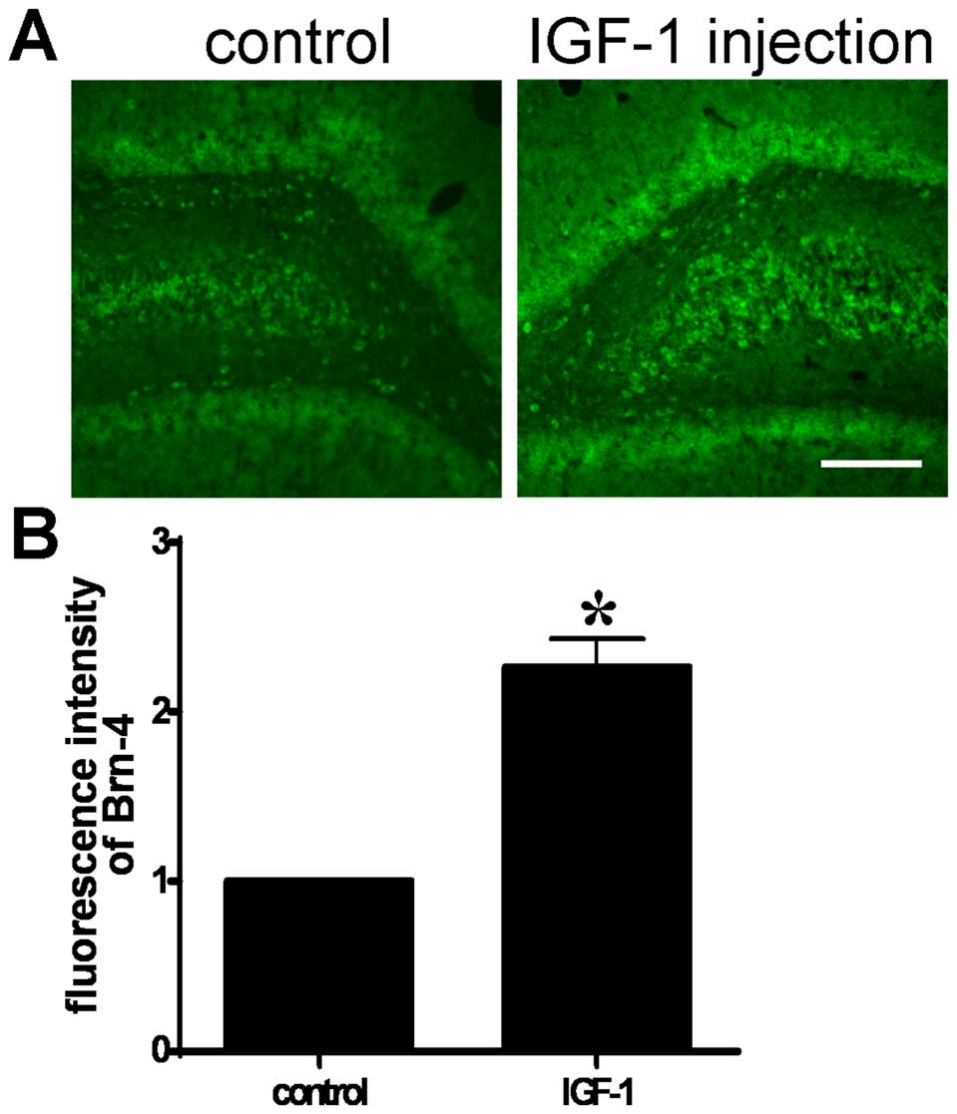
IGF-1 increases Brn-4 expression in adult hippocampus *in vivo*. IGF-1 (0.5 µg/100 g body weight) was injected to the right side hippocampus of normal adult rat. Three days later, coronal sections (20 µm) of the hippocampus were prepared. (A) Immunofluorescence staining with Brn-4 antibody of a coronal section through hippocampus. Scale bar, 300 µm. (B) Quantification of immunofluroscence intensity in (A). The left side (control) set to 1. Data are expressed as means ± SEM (n = 3). *, *P*<0.05 compared with control.

The increase in the levels of IGF-1 following denervation raised the question of whether IGF-1 might be responsible for the increased expression of Brn-4 seen after FiFx transection. We therefore investigated the effect of IGF-1 on Brn-4 expression. Firstly, IGF-1 was injected to the right side hippocampus of normal adult rat. Three days later, we compared the Brn-4 levels in the injected hippocampus with the contraisolateral side. The coronal sections through hippocampus were stained with Brn-4 antibody. Analysis of the immunofluorescence intensity showed that IGF-1 increased significantly Brn-4 expression in hippocampus (Fig. 3A and B).

**Figure 4:**
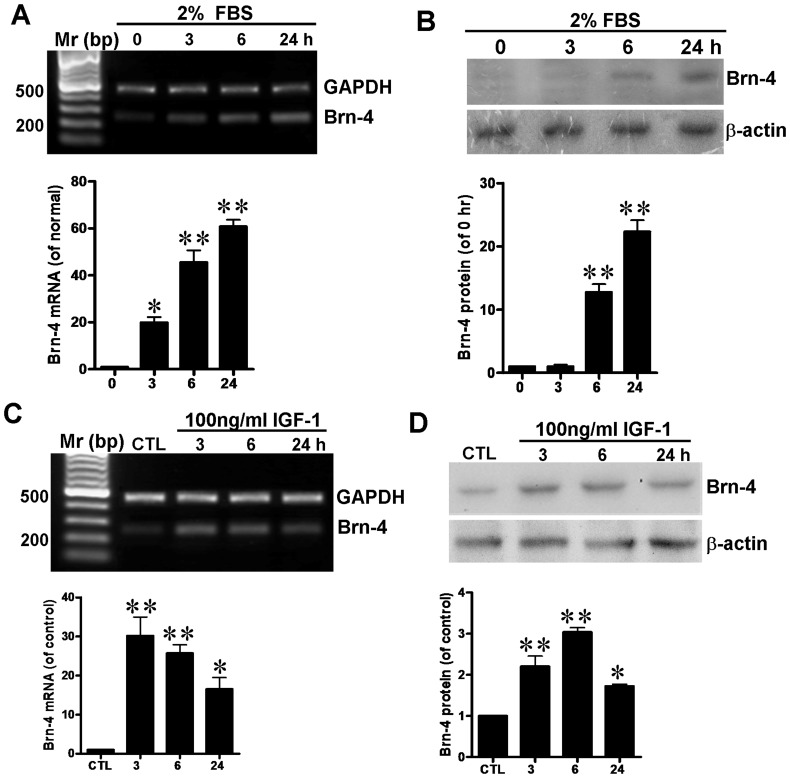
Brn-4 expression in hippocampus-derived NSCs cultured in differentiation medium with (C, D) or without (A, B) IGF-2. (**A**) Brn-4 mRNA levels in hippocampal NSCs determined by RT-PCR at different time-points (0, 3, 6, and 24 h) following transfer to differentiation medium (DMEM, 2% FBS). GAPDH was used as the reference. Lower panel is quantification of Brn-4 mRNA. (**B**) Brn-4 protein (39 kDa) levels detected by western blotting different time-points (0, 3, 6, and 24 h) following transfer to differentiation medium; β-actin (42 kDa) provided the reference. Lower panel is quantification of Brn-4 protein. Data are means ± s.e.m.; *, *P*<0.05; **, *P*<0.01 versus 0 h. (**C, D**) Effect of IGF-1 on Brn-4 mRNA and protein expression in hippocampal NSCs *in vitro*. Hippocampal NSCs were incubated in differentiation medium containing IGF-1 (100 ng/ml) for 3, 6 or 24 h; control cells were incubated in differentiation medium alone for 24 h. Brn-4 mRNA and protein were determined by RT-PCR (C) and western blotting (D) analysis, demonstrating upregulation of Brn-4 after IGF-1 treatment in time-course manner. Data are means ± s.e.m.; *, *P*<0.05; **, *P*<0.01 versus control (CTL).

*In vitro*, hippocampus-derived NSCs were transferred to differentiation medium (DMEM containing 2% FBS). At 0, 3, 6 or 24 h after culture, levels of Brn-4 were determined using RT-PCR and western blotting analysis. Exposure of hippocampal NSCs to differentiation medium increased Brn-4 transcription in a time-dependent manner (Fig. 4A), and there was a parallel increase in levels of Brn-4 protein (Fig. 4B). We then examined whether supplementation of differentiation medium with IGF-1 would affect the extent or the kinetics of Brn-4 expression. Hippocampal NSCs were transferred to differentiation medium containing IGF-1 (100 ng/ml) as described by others [16], [17], [18] for 3, 6 or 24 h. Control hippocampal NSCs were cultured only with differentiation medium for 24 h. As shown in Fig. 4C, and D, supplementation with IGF-1 brought a rapid increase in levels of Brn-4 mRNA determined by RT-PCR, with a marked upregulation at 3 h versus differentiation medium alone (compare Fig. 4C with Fig. 4A). At later time-points there was a fall in the levels of Brn-4 levels in IGF-1-treated NSCs versus cells treated with differentiation medium alone (Fig. 4C versus Fig. 4A). At the protein level there was an even more marked acceleration of Brn-4 expression. Western blotting revealed that Brn-4 protein in hippocampal NSCs was significantly increased and reached a peak 6 h after treatment with IGF-1, and decreased again at 24 h (Fig. 4D), whereas peak protein levels in cells treated with differentiation medium alone were only seen at 24 h (compare Fig. 4D with Fig. 4B).

Together, these results demonstrate that IGF-1 markedly accelerates the expression of Brn-4, at both the mRNA and protein levels, versus expression in differentiation medium alone. This finding is broadly consistent with a report that IGF-1 increased Brn-4 expression in striatal NSCs [16].

### 4. IGF-1 induces neuronal differentiation of hippocampal NSCs *in vitro*

**Figure 5:**
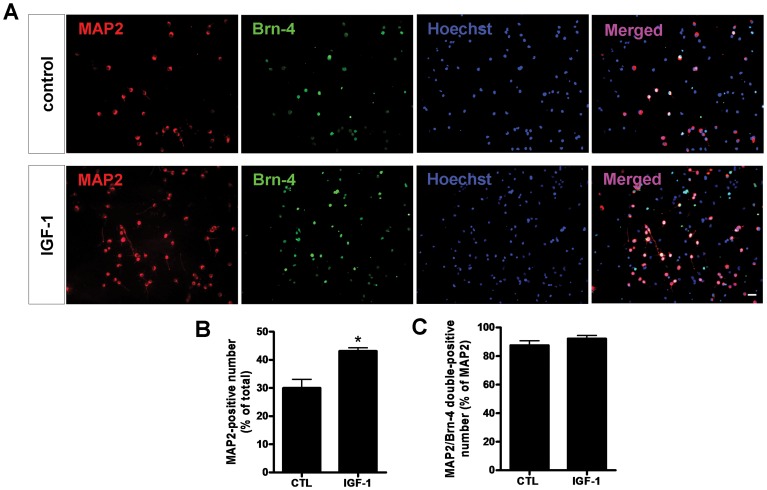
Immunofluorescence analysis of Brn-4 expression and neuronal differentiation (MAP2 expression) of hippocampal NSCs after treatment with (IGF-1) or without (control) 100 ng/ml IGF-1. (**A**) Cells were stained separately for MAP2, Brn-4 and the total cells were indicated by Hoechst staining. Scale bar, 25 µm. (**B**) Quantification of MAP2 positive neurons as a percentage of total cells. (**C**) Quantification of MAP2/Brn-4 double-positive neurons as a percentage of MAP2-positive neurons. Data are means ± s.e.m of 10 randomly selected microscopic fields in each experiment (*n* = 3). *, *P*<0.05 versus control.

Because IGF-1 accelerates the expression of Brn-4, we determined whether IGF-1 might similarly expedite the differentiation of NSCs into neurons. Hippocampal NSCs were seeded onto poly-L-lysine-coated coverslips and transferred to differentiation medium with or without IGF-1 (100 ng/ml) for 6 h, followed withdraw of the factor treatment. After further 5-day culture, the neuronal differentiation of hippocampal NSCs was examined by immunofluorescence assay using antibody against MAP2, a marker of neuronal differentiation (Fig. 5A).

In the control group, 29.99±5.39% of total cells were MAP2-positive, indicative of neuronal differentiation (Fig. 5A, B). By contrast, in the IGF-1-treated cultures 43.10±1.99% of cells were MAP2-positive (*P*<0.05) (Fig. 5A, B). To confirm the association between Brn-4 expression and neuronal differentiation under both conditions, cultures were examined for fluorescence with an anti-Brn-4 antibody. As shown in Fig. 5A, in control cultures 87.61±5.38% of MAP2-positive cells were also Brn-4-positive in control cells, whereas in IGF-1-treated cultures 92.23±3.81% of MAP2 positive cells were also positive for Brn-4 (*P*>0.05). These results indicate that neuronal differentiation is accompanied by Brn-4 expression under both conditions, but that IGF-1 treatment promotes both Brn-4 expression and neuronal differentiation of hippocampal NSCs.

### 5. PI3K/Akt mediates IGF-1 induced Brn-4 expression *in vitro*

**Figure 6:**
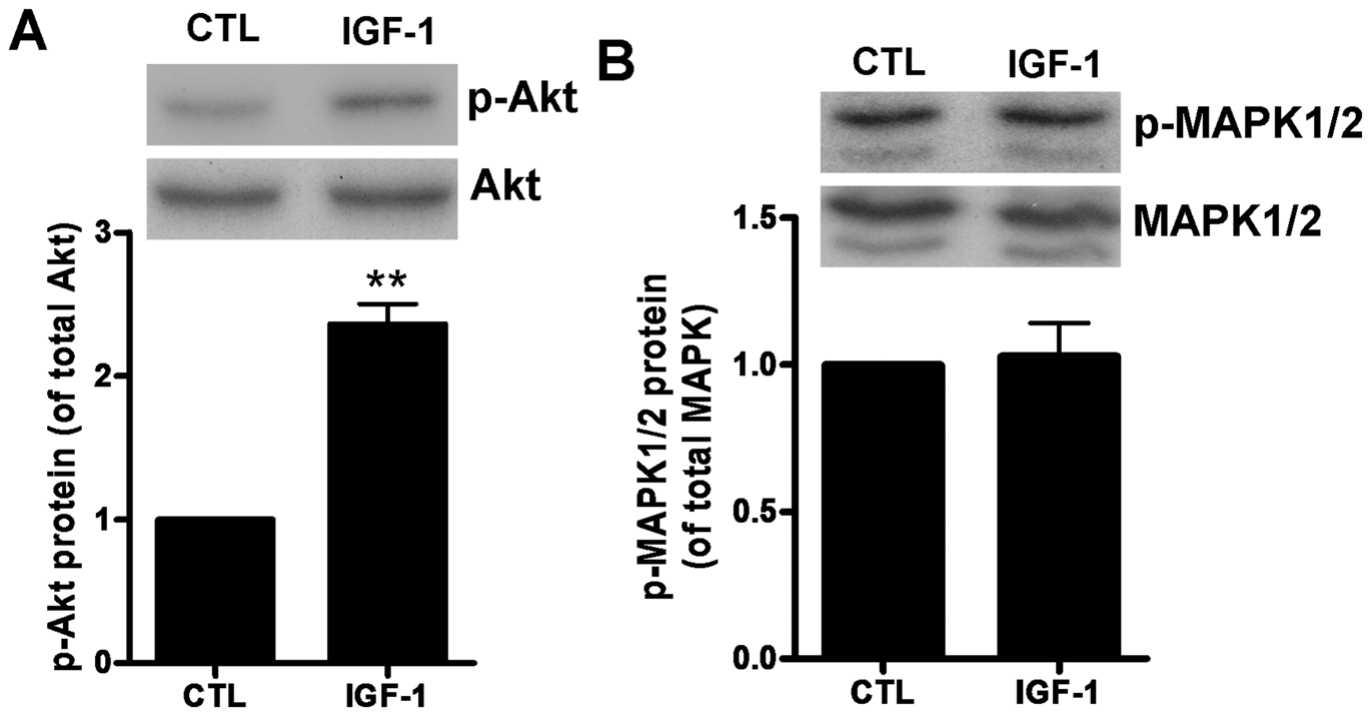
IGF-1 promotes Brn-4 expression through PI3K/Akt pathway. (**A, B**): Hippocampal NSCs were transferred to differentiation medium with (IGF-1) or without (control) 100 ng/ml IGF-1 for 6 h. Cell lysates were analyzed by western blotting using antibodies specific for Akt, phospho-Akt (p-Akt), MAPK1/2, or phospho-MAPK (p-MAPK1/2). (**A**) Upper: Western blotting assay for p-Akt and total Akt protein showing in increase in p-Akt levels but no change in Akt protein levels; Lower: Quantification of the upper image in (A) expressed as p-Akt/Akt ratios. (**B**) Upper: Western blotting for p-MAPK1/2 and total MAPK1/2 protein; Lower: Quantification of the upper image in (B) expressed as p-MAPK/MAPK ratios. Data are means ± s.e.m. **, *P*<0.01 compared to control (CTL).

IGF-1, acting via the IGF-1 receptor (IGF1R), is known to regulate diverse aspects of cellular physiology via the phosphoinositide 3-kinase (PI3K)–Akt pathway; however, other pathways such as via the MAP kinase (MAPK/ERK) kinase MEK have also been implicated in some aspects of IGF-1 signaling. PI3K is thought to act via phosphorylation and activation of Akt. To determine the signaling pathway underlying IGF-1-induced Brn-4 expression in NSCs, we examined the levels of phospho-Akt (p-Akt) and phospho-MAPK (p-MAPK) in hippocampal NSCs treated with IGF-1 (100 ng/ml, 6 h). Western blotting analysis revealed that total Akt protein levels were unaffected by IGF-1 treatment, whereas p-Akt levels were increased by over twofold (Fig. 6A). Confirming the specificity of the phosphorylation, IGF-1 treatment was without any effect on either phosphorylation or protein levels of MAPK (Fig. 6B).

**Figure 7:**
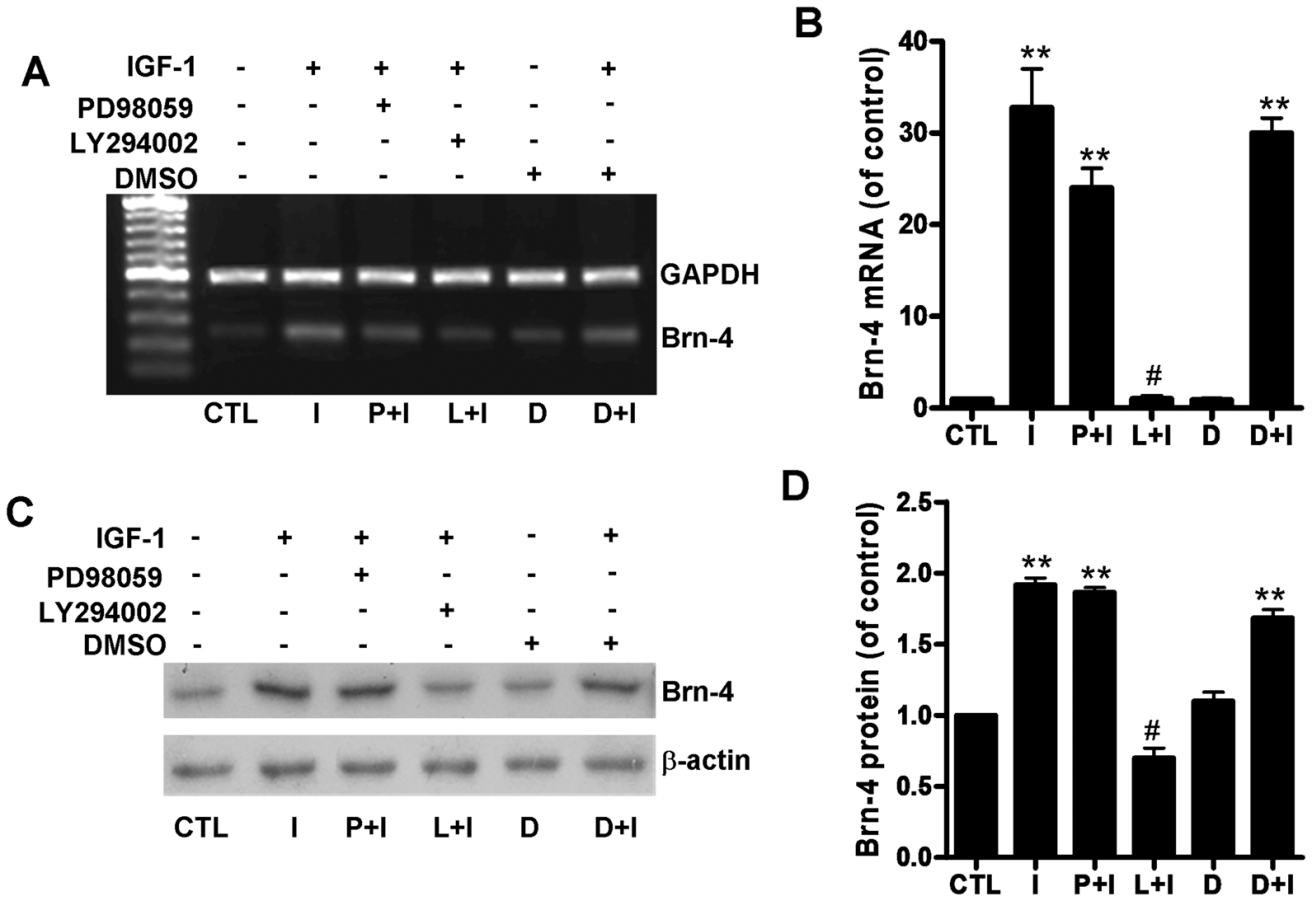
PI3K inhibition attenuates IGF-1-induced Brn-4 upregulation in hippocampal NSCs. Cells were pretreated with PD98059, LY294002, or DMSO vehicle for 40 min and then stimulated by IGF-1 for 6 h. (**A, B**) Brn-4 mRNA levels determined by RT-PCR, GAPDH was used as a reference. (**C, D**) Western blotting analysis using Brn-4 and β-actin (reference) antibodies. Data are expressed as means ± SEM. **, *P*<0.01 compared with control; #, *P*<0.01 versus the IGF-1 group. Abbreviations: D, DMSO. I, IGF-1; L, LY294002; P, PD98059.

We therefore employed selective inhibitors of PI3K (LY294002) or MEK (PD98059) to determine if these pathways are involved in IGF-1-mediated upregulation of Brn-4 and neuronal differentiation. NSCs were transferred to differentiation medium containing the following supplements: (1) no addition, (2) IGF-1 (100 ng/ml), (3) IGF-1 (100 ng/ml) plus PD98059 (50 µM), (4) IGF-1 (100 ng/ml) plus LY294002 (50 µM) (5) DMSO (1%), and (6) DMSO (1%)+IGF-1 (100 ng/ml). In all cases treatment with either inhibitor (PD98059 and LY294002) or vehicle (DMSO) was commenced 40 min [17], [18] before addition, where appropriate, of IFG-1. After 6 h of IGF-1 treatment, total protein and RNA were extracted and examined for Brn-4 expression using RT-PCR and western blotting. As shown in Fig. 7, IGF-1 produced a significant increase in Brn-4 mRNA levels assessed by RT-PCR. Pretreatment with either the MEK inhibitor PD98059 or DMSO vehicle did not affect Brn-4 expression. By contrast, treatment with the PI3K inhibitor LY294002 markedly attenuated the IGF-1-mediated Brn-4 upregulation of NSCs (Fig. 7A, B). A similar profile was seen at the protein level, where IGF-1 treatment led to a twofold increase in Brn-4 polypeptide levels. The increase was unaffected by MEK inhibitor PD98059 or DMSO, whereas the IGF-1-mediated increase in Brn-4 protein levels was abrogated by PI3K inhibition with LY294002 (Fig. 7C, D).

**Figure 8:**
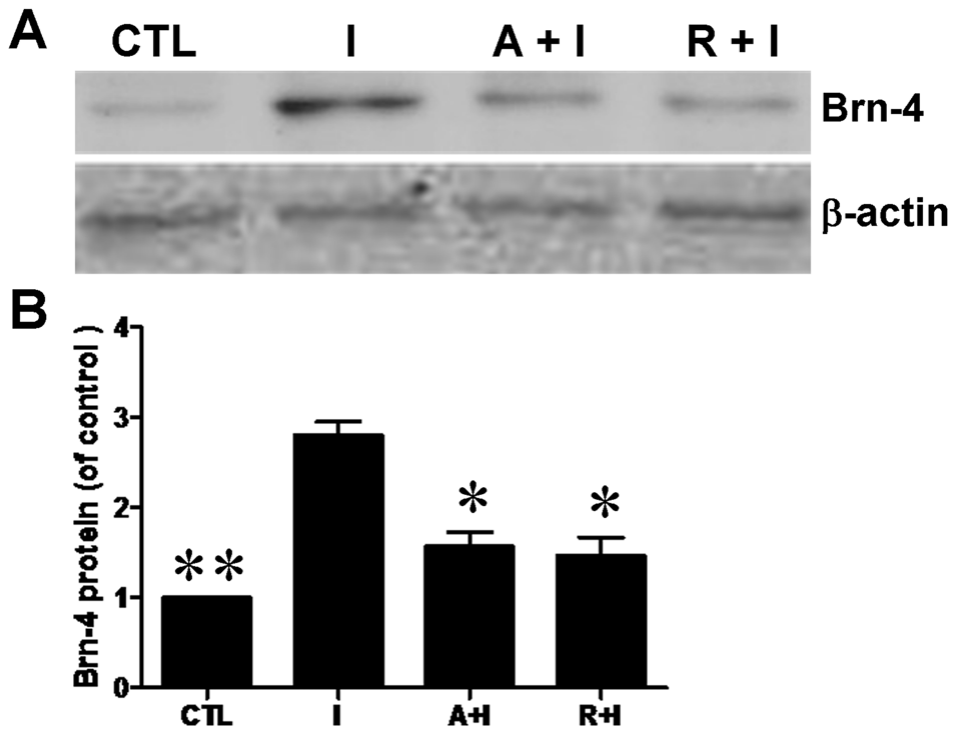
Increase of Brn-4 induced by IGF-1 is attenuated by both AG1024 and rapamycin. Hippocampal NSCs were pretreated with AG1024 or rapamycin for 1 h and subsequently stimulated with IGF-1 for 6 h, and then cell lysates were analyzed by western blotting using antibody for Brn-4. Upper: Western blotting assay for Brn-4 and β-actin. Lower: Quantification of the upper image expressed as Brn-4/β-actin ratio. Data are means ± s.e.m. *, P<0.05 and **, *P*<0.01 compared to IGF-1 group. Abbreviations: I, IGF-1; A, AG1024; R, rapamycin.

Mammalian target of rapamycin (mTOR) is reported to be a downstream signal molecule of PI3K/Akt and play an important role in the differentiation of several types of cells [19], [20]. In order to further clarify the pathway through which IGF-1 induces the differentiation of hippocampal NSCs, we used AG1024, inhibitor of IGF-1 receptor and rapamycin, inhibitor of mTOR. Hippocampal NSCs were pretreated with AG1024 (10 µM) or rapamycin (5 nM) for 1 h, and then IGF-1 (100 ng/ml) was added to the medium for 6 h. The lysates were collected for western blotting analysis. As shown in Fig. 8, both rapamycin and AG1024 could attenuate the increased expression of Brn-4 induced by IGF-1. Thus, we conclude that IGF-1 is likely to promote Brn-4 expression (and by inference neuronal differentiation) of hippocampal NSCs by acting via the PI3K/Akt signaling pathway *in vitro*.

### 6. PI3K/Akt mediates IGF-1 induced neural differentiation

**Figure 9:**
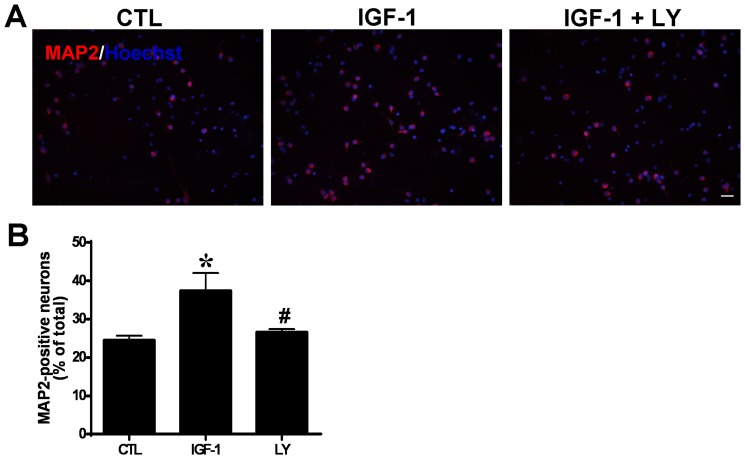
PI3K inhibition decreases IGF-1-induced neuronal differentiation of hippocampal NSCs. Cells were pretreated with LY294002 or DMSO for 40 min and then stimulated by IGF-1. (**A**) MAP-2 immunofluorescence analysis of neuronal differentiation of hippocampal NSCs. Nuclei were counterstained with Hoechst (blue). Scale bar, 25 µm. (**B**) Quantification of the MAP2-positive neurons in (**A**), Data are expressed as means ± SEM. **, *P*<0.01 compared with control; #, *P*<0.01 versus the IGF-1 group. LY, LY294002.

To make sure whether PI3K/Akt pathway is involved in the IGF-1 induced neural differentiation of hippocampus-derived NSCs, the cells were pretreated with inhibitors of PI3K, LY294002 (20 µM) for 2 hours, followed with IGF-1 treatment. 5 days later, neural differentiation was assessed using MAP2 immunostaining. The result showed that IGF-1 induced neural differentiation was significantly reduced by LY294002 pretreatment (Fig. 9), suggesting that PI3K/Akt signal pathway is involved in the neural differentiation of the hipppocampal NSCs when induced by IGF-1.

## Discussion

Because of their neuronal differentiation ability, NSCs provide a potential source of cells for replacement therapy in neurodegenerative disease [21], [22]. However, the low efficiency of *in vitro* neuronal differentiation of NSCs is so far inadequate to meet therapeutic demands [3], [4], [5]. There is thus an urgent need to explore the pathways and mechanisms involved in neuronal differentiation to produce sufficient neurons/neural precursors to meet the needs of clinical treatment.

In our previous studies we reported that FiFx transection robustly induced the proliferation, migration, and neuronal differentiation of engrafted or endogenous NSCs in the hippocampal dentate gyrus [6], [7]. Neuronal differentiation was accompanied by upregulation of hippocampal Brn-4 expression, and gain or loss of function experiments have argued that Brn-4 plays a positive role in the neuronal differentiation of hippocampus-derived NSCs [9], [15]. However, the underlying pathways or mechanisms remain unclear. Shimazaki *et al.* [16] reported that exposure of NSCs derived from the E14 mouse striatum to IGF-1 and BDNF resulted in rapid upregulation of Brn-4 mRNA and protein. IGF-1 is predominantly produced by the liver, but is also expressed in neurons and glia [23], [24] where it is known to play an important role in normal brain development, promoting neuronal growth, cellular proliferation and differentiation [25], [26], [27], [28], [29]. Learning and memory in adult rats are promoted by an IGF-1-dependent mechanism related to hippocampal neurogenesis [30], [31]. BDNF stimulates the formation and increases of dendritic spines in hippocampal neurons [32], [33], [34] and BDNF can induce neuronal differentiation, synaptic plasticity [35], [36], and neuroprotection [37], [38]. We thus hypothesized that IGF-1, and perhaps also BDNF, might be involved in hippocampal neurogenesis following denervation damage, and might therefore promote the survival and differentiation of hippocampal NSCs.

In the present study we demonstrated that *in vivo* levels of IGF-1 mRNA and protein in the hippocampus (but not BDNF; data not shown), were upregulated at day 7 following surgical FiFx transection. Treatment *in vitro* of NSCs derived from E14 rat hippocampus with IGF-1 (100 ng/ml) caused significant increases in Brn-4 mRNA and protein expression. Moreover, the number of cells positive for the neuronal marker MAP2 was also increased by IGF-1 treatment.

Overall, our previous and present results suggest a cause/effect cascade in which hippocampal denervation damage induces IGF-1 expression in hippocampus which, in turn, promotes local Brn-4 expression that is thought to drive neuronal differentiation of NSCs. The increase of Brn-4 expression after IGF-1 treatment may be due to the more neuronal differentiation induced by IGF-1. We also found that a small number MAP2-negative cells expressed Brn-4; because Shimazaki *et al.* [16] observed low levels of Brn-4 expression in some, but not all, astroglial cells, we speculate that these MAP2-negative Brn-4-positive cells may be glial cells. Other members of POU class III genes are expressed in both neuronal and glial cells in the developing central nervous system [39], [40]. Therefore, it is possible that Brn-4 also plays a role in glial cell development, although the results of our experiments suggest that this role may be minor.

**Figure 10:**
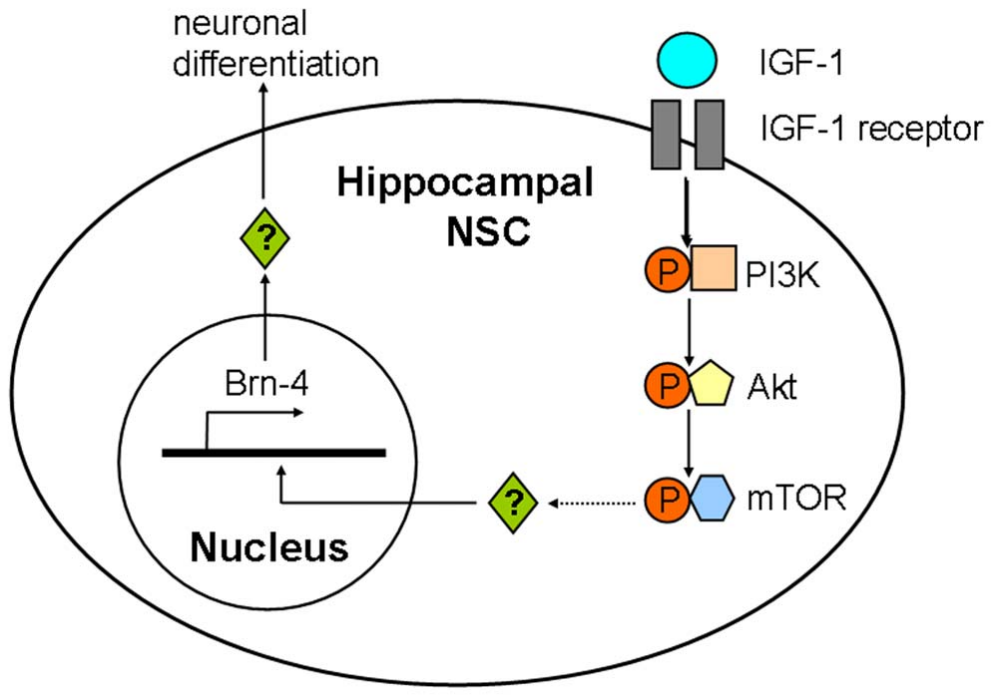
Schematic diagram of the proposed pathway through which IGF-1 induces expression of Brn-4 and neuronal differentiation of hippocampal NSCs. IGF-1 binds to its receptor in the membrane of NSCs. Then PI3K/Akt are phosphorylated. Subsequently, mTOR, the downstream signal molecule of PI3K/Akt, is activated. As found in this study, mTOR mediated the expression of Brn-4 and neurogenesis function of hippocampal NSCs induced by IGF-1, whereas its downstream effectors are needed to be clarified further.

Depending on cell type and context, IGF-1 is known to promote neuronal survival, maturation [41], [42], [43], [44], [45], and cell-cycle progression [46] by activating the PI3K/Akt and/or Ras/MAPK pathways. Using previously established protocols [16], [17], [18], we investigated whether inhibition of either the PI3K or MAPK pathways would interfere with IGF-1-induced upregulation of Brn-4. We clarified that cells treated with the PI3K inhibitor LY294002 failed to upregulate Brn-4 in response to IGF-1, whereas Brn-4 upregulation was unaffected by treatment with the MAPK inhibitor PD98059. In addition, we found that both AG1024 and rapamycin could attenuate the increased expression of Brn-4 induced by IGF-1. Thus, as shown in Fig. 10, we speculate that extracellular IGF-1 binds to the IGF-1 receptor in the membrane of hippocampal NSCs, then intracellular PI3K/Akt and their downstream signal molecule, mTOR, are activated in sequence. Combined with our previous findings, we think these changes will lead to increased expression of Brn-4, and ultimately neuronal differentiation of NSCs. However, the reactions in nucleus caused by these changes remain to be addressed. Interestingly, various inhibitors used in our experiments did not completely inhibit the expression of Brn-4. These phenomena may be related to time and dose of inhibitors. Of course, we can not deny that except for PI3K/Akt pathway, other pathways may also participate in the process of Brn-4 expression induced by IGF-1. Additionally, we clarified that exogenous IGF-1 can stimulate Brn-4 expression in the adult rat hippocampus. But we didn’t test whether IGF-1 effect Brn-4 expression *via* PI3K/Akt pathway *in vivo*. All of these need to be further demonstrated. In a word, we report that Brn-4 expression induced by IGF-1 is dependent on the PI3K/Akt pathway *in vitro*, but not on the Ras/MAPK pathway.

It has previously been reported that PI3K/Akt signaling pathway plays an important role in neural cell proliferation and differentiation [47], [48], [49]. Moreover, this pathway is required in the process of IGF-1-induced neural progenitor proliferation and differentiation [50], [51], [52]. In addition, Lin *et al.* [53] reported that administration of IGF-1 could attenuate hypoxic–ischemic brain injury in neonatal rats acting via the PI3K/Akt pathway leading to inhibition of apoptotic cell death. However, these results contrast with those of Shimazaki [16] who have demonstrated that either IGF-I or BDNF resulted in a rapid upregulation of Brn-4 that mediates differentiation of striatal neuron-precursor, although it is possible that this apparent discrepancy may be explained by the use of different cell species.

The overall picture emerging is that, following brain injury such as FiFx lesion, damage leads to upregulation of IGF-1 which, acting via the PI3K/Akt signaling pathway, in turn leads to upregulation of the key transcription factor Brn-4, thereby promoting NSCs differentiation along neuronal pathways. By this mechanism, IGF-1 expression in the lesioned hippocampus would provide a microenviroment for the survival and differentiation of NSCs *in vivo*. We surmise that these changes are likely to underlie the processes of hippocampal repair and neurogenesis after injury. Although the mechanisms underlying this process have not yet been fully elucidated *in vivo*, our results provide a theoretical basis for promoting neuronal differentiation of hippocampal NSCs *in vitro*. These findings have potential in the development of NSCs for the clinical treatment of neurological disease.
